# Unravelling the interfacial water structure at the photocatalyst strontium titanate by sum frequency generation spectroscopy†

**DOI:** 10.1039/d3cp03829g

**Published:** 2023-10-31

**Authors:** Martin Buessler, Shingo Maruyama, Moritz Zelenka, Hiroshi Onishi, Ellen H.G. Backus

**Affiliations:** a University of Vienna, Faculty of Chemistry, Institute of Physical Chemistry, Währinger Straße 42 1090 Vienna Austria ellen.backus@univie.ac.at; b University of Vienna, Vienna Doctoral School in Chemistry (DoSChem), Währinger Straße 42 1090 Vienna Austria; c Department of Applied Chemistry, Graduate School of Science, Tohoku University Sendai Miyagi Japan; d Department of Chemistry, School of Science, Kobe University, Rokko-dai, Nada Kobe Japan; e Division of Advanced Molecular Science, Institute for Molecular Science, Myodaiji Okazaki Japan

## Abstract

The direct conversion of solar energy to hydrogen is considered as a possible method to produce carbon neutral hydrogen fuel. The mechanism of photocatalytic water splitting involves the chemical breakdown of water and re-assembly into hydrogen and oxygen at the interface of a photocatalyst. The selection rules of a suitable material are well established, but the fundamental understanding of the mechanisms, occurring at the interface between the catalyst and the water, remains missing. Using surface specific sum frequency generation spectroscopy, we present here characterisation of the interface between water and the photocatalyst strontium titanate (SrTiO_3_). We monitor the OH-stretching vibrations present at the interface. Their variations of intensities and frequencies as functions of isotopic dilution, pH and salt concentration provide information about the nature of the hydrogen bonding environment. We observe the presence of water molecules that flip their orientation at pH 5 indicating the point of zero charge of the SrTiO_3_ layer. These water molecules are oriented with their hydrogen away from the surface when the pH of the solutions is below 5 and pointing towards the surface when the pH is higher than 5. Besides, water molecules donating a H-bond to probably surface TiOH groups are observed at all pH.

## Abstract 2

Unravelling the water structure at the interface between the photocatalyst strontium titanate and water by sum frequency generation spectroscopy.

## Graphical abstract

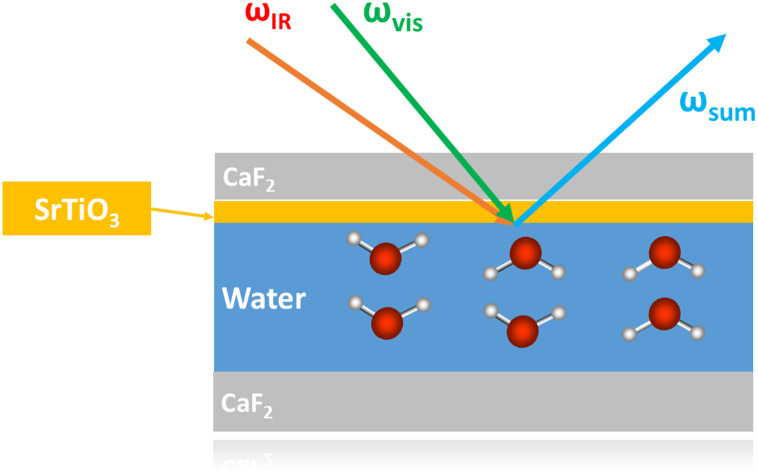

## 1. Introduction

Currently, 98% of hydrogen is produced from fossil resources and only 2% from renewable energy sources.^1^ Discovered in 1972, the production of hydrogen by photocatalytic water splitting is one of the most promising approaches to the large-scale conversion of solar energy into hydrogen.^2^ This approach involves the conversion of photonic energy into chemical energy with a semiconductor as a catalyst. Its band gap should ideally be around 2.1 eV and its conduction and valence band potential should be on both sides of the potential needed for the water oxidation (−1.23 eV *vs*. a standard hydrogen electrode (SHE)) and water reduction (0 eV *vs*. SHE) reactions to occur. Moreover, its lattice structure should allow a long life time of the photogenerated charges and it should be an abundant and non-toxic material.^3^ Only a few materials have been found to fulfill all those specifications, *e.g.*, TiO_2_,^4^ WO_3_,^5^ SrTiO_3_,^6,7^ NaTaO_3_,^8^ and M_*x*_Fe_3−*x*_O_4_.^9^

However, even knowing the prerequisite to select a photocatalyst able to drive the water splitting reactions, this is not sufficient to optimize and to design an efficient material. Insights into the chemical reactions at the molecular level during water oxidation and water reduction are also important but are currently very scarce. Investigating the dynamics of water at the interface with the catalysts during water splitting reactions remains challenging and would provide prominent knowledge about how the water structurally interacts with the catalyst surface. As a first step, we look at the static water structure at the interface with the photocatalyst strontium titanate (SrTiO_3_/STO).

STO has high chemical and thermal stability, and its strong photocatalytic activity under UV light has attracted much interest. Its capacity, as a perovskite material, to be tuned by dopant elements, is seen as a major advantage in developing an efficient STO based water splitting catalyst.^10^ Previous works have theoretically^11–13^ and experimentally^14–16^ investigated the interaction between water and STO. In contact with water below 300 K, the strontium atoms are expected to dissolve resulting in TiO_2_-terminated SrTiO_3_ surfaces.^11,15^ Surface X-ray diffraction studying the effects of a drop of liquid water on a strontium titanate surface showed that the STO surface exhibits large terraces composed of either TiO_2_ or SrO.^14^ More specifically, density functional theory and photoemission spectroscopy studying the interaction of water vapour with STO under ultrahigh vacuum have reported that the SrTiO_3_ surface consists of a two-dimensional titania layer composed of TiO_4_ tetrahedra.^16^ To date, the available data suggested that the STO surface is similar to a titania terminated surface, but no experimental work considering a macroscopic amount of water at the interface with the STO under ambient conditions has been reported.

At such an interface, molecular specific interfacial information could be obtained by using vibrational sum frequency generation (SFG) spectroscopy.^17^ In SFG spectroscopy an infrared beam in resonance with a molecular vibration and a visible laser beam are combined at the interface which then leads to the generation of light at the sum frequency. As no sum frequency light is generated in centrosymmetric media, like bulk water, this method provides information about the water alignment at the interface and the frequency of the resonance reports on the hydrogen bonding strength.^18^ SFG spectroscopy has successfully been used to study water at the interface with, *e.g.*, calcium fluoride,^19,20^ silica,^21^ alumina^22^ and mica.^23^ Recently, this technique has been applied to determine the water structure at the TiO_2_–water interface for both a thin amorphous layer^24,25^ and an anatase film.^26^

In this work, we use SFG spectroscopy to investigate the water structure in the presence of a macroscopic amount of water at the interface with polycrystalline STO films of 100 nm thickness deposited on a CaF_2_ substrate. As interfacial charges might be relevant for photocatalytic water splitting, we study the interface at different bulk pH values resulting in protonation (low pH) and deprotonation (high pH) of the STO layer. Moreover, we perform experiments at different salt concentrations and isotopic dilutions to distinguish the different water species observed at the interface in acidic and alkaline environments.

## 2. Experimental section

### 2.1. SFG setup

**Fig. 1:**
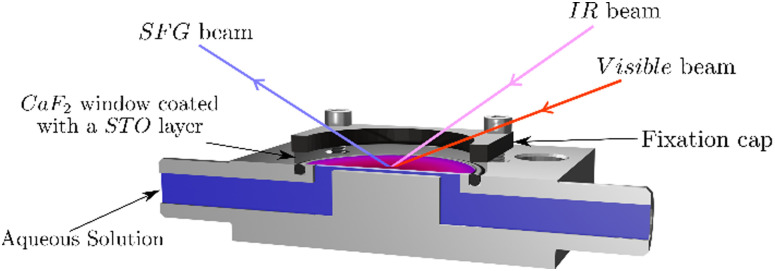
Sketch of the experimental cell. Both IR and visible beams are overlapped in time and space at the interface between the STO layer and the water. The SFG beam is reflected through the CaF_2_ substrate out to the detection path. The aqueous solution is pumped in and out of the cell without unmounting the sample: which allows successive experiments to be performed on the same sample position.

The SFG setup is based on a femtosecond Ti-sapphire laser (Libra, Coherent). It has an output power of 5 W at 800 nm with a repetition rate of 1 kHz and a pulse duration of about 50 fs. Part of the laser beam is guided through a Fabry–Pérot interferometer in order to narrow its spectral width to about 1 nm. Another part of the laser beam is guided through an optical parametric amplifier (TOPAS PRIME, Light Conversion) and through a non-collinear DFG-stage generating an infrared beam of 3.9 μJ centred at 3200 cm^−1^ with a full-width-half-maximum (FWHM) of about 340 cm^−1^. Those two beams are focused and overlapped in space and time at the interface between the STO layer and water generating the SFG signal, see Fig. 1. The angles of incidence are 41° for the IR and 63° for the visible with respect to the surface normal. The SFG signal is reflected from the solid–liquid interface and guided to a spectrometer (Shamrock 303i, Andor) to be detected using a CCD chip (EMCCD-Detector, Newton) working at −75 °C. To correct for the frequency dependent intensity of the IR beam, in certain experiments, the spectrum is normalised to a spectrum from a gold coated CaF_2_ window.^19^

The laser source for the phase-resolved SFG spectroscopy measurements is a Ti:sapphire amplifier (Astrella, Coherent) with a 1 kHz repetition rate, *ca.* 40 fs pulse duration and a pulse energy of 7 mJ. The 800 nm output pulse is split into several parts. 2 mJ are used to generate the IR beam for the experiments using an optical parametric amplifier (TOPAS PRIME, Light Conversion) with a successive NDFG stage (Light Conversion). The resulting IR beam has an energy of about 4 μJ centred at 3200 cm^−1^ and a FWHM of about 300 cm^−1^. The visible beam is produced by sending 1 mJ of the laser output through a pulse shaper to narrow the pulse further down to a FWHM of about 1.4 nm and an energy of about 20 μJ. The visible and IR pulses are then focused on the local oscillator with a fused silica lens (LA4716-B, Thorlabs) and a CaF_2_ lens (LA5042, Thorlabs), respectively. The local oscillator is a 100 nm layer of ZnO sputtered on a CaF_2_ substrate (2 mm × 25 mm, Crystal GmbH). The visible, IR and SFG_LO_ beams are then collimated by a 60° off-axis parabolic mirror (MPD246-P01, Thorlabs). The SFG_LO_ light is passed through a 1 mm thick glass plate to induce a time delay in the pulse. The beams are then refocused onto the sample by a 45° off-axis parabolic mirror (MPD284-P01, Thorlabs). The incident angles for the visible and IR beam are ∼35° and ∼25° with respect to the sample surface normal, respectively. The reflected SFG signals of the sample and local oscillator are collimated and refocused onto the slit of a spectrometer (IsoPlane 160, grating: 1800 grooves per mm, Princeton Instruments) and detected with an EMCCD camera (ProEM, Princeton Instruments). The cooling of the EMCCD chip is set to −60 °C. The obtained spectra were processed as described in the literature.^27^ Briefly, the measured spectrum is inverse Fourier-transformed from the frequency domain to the time domain. A filter function is applied to select one of the cross terms, which is then Fourier-transformed back to the frequency domain. The same procedure is applied to the reference spectrum. The sample spectrum is then divided by the reference spectrum to get the normalized phase-resolved spectrum. It was not necessary to apply a correction for the phase drift between the local oscillator and the sample signal.

### 2.2. Sample preparation

Polycrystalline STO thin films of 100 nm were grown on a calcium fluoride (CaF_2_) substrate by pulsed laser deposition. The substrate is a CaF_2_ window of 25 mm diameter and 2 mm thickness (Crystal GmbH, Berlin, Germany). The STO (111) single crystal substrate (Shinkosha Co., Ltd, Japan) was used as a target and KrF excimer laser (*λ* = 248 nm) ablation was carried out at a substrate temperature of 700 °C under an oxygen pressure of 1 × 10^−5^ Torr. The laser fluence and repetition rate were 0.5 J cm^−2^ and 2 Hz, respectively. The obtained thin films were characterized by X-ray diffraction and atomic force microscope (see the ESI† Fig. S1 and S2).

### 2.3. Solution preparation

Solutions with pH values ranging from pH 3 to pH 11 were prepared using HCl (Sigma Aldrich, 37%) and NaOH (Sigma Aldrich, 98.5%) and ultrapure water (Merck MilliQ, 18.2 MOhm cm at 25 °C). Furthermore, the ionic strength of the solution was always adjusted with NaCl (Sigma Aldrich, 99.0%) to 1 mM, if needed. All pH values were measured with a pH-meter (Mettler-Toledo) to an accuracy of 0.1. Salt solutions were prepared with ultrapure water and additional NaCl (Sigma Aldrich, 99.0%). Isotopic dilutions were prepared of pH 3 and pH 11 with a mixture of H_2_O and D_2_O (Eurisotop, 99.9%) solution.

### 2.4. Sample cell mount

As the STO layers are not fully homogeneous, we use a flow cell to ensure to measure the same STO spot upon changing the aqueous solutions. Fig. 1 shows the different beams guided through the CaF_2_ window and the aqueous solution pumped into contact with the STO layer. Between each solution, the cell is flushed with dry-air and washed with ultrapure water. The pump used is an analog L/S Masterflex equipped with Tygon tubes (*ϕ*: 4.8 mm, Ismatec).

## 3. Results and discussion

**Fig. 2:**
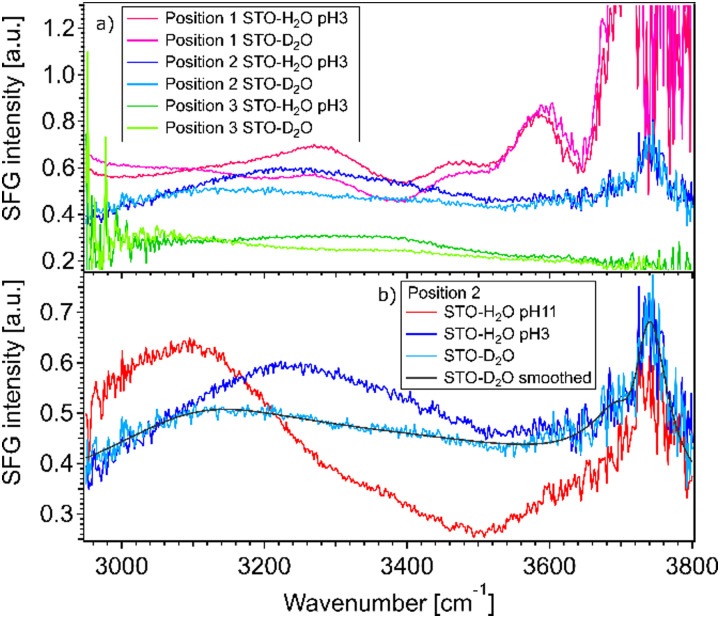
(a) Spectra for water pH 3 and pure D_2_O (pD 7), normalized with gold, in contact with a 100 nm SrTiO_3_ layer at three different positions on the sample. (b) Spectra for pure D_2_O (pD 7), water pH 3 and water pH 11, normalized with gold, in contact with a 100 nm SrTiO_3_ layer at position number 2 on the sample. The smoothed STO–D_2_O curve is depicted in black (= *A*_NR_(ω)^2^).

Fig. 2(a) shows SFG spectra at three different sample positions obtained for STO–water pH 3 and STO–D_2_O interfaces. In the case of STO–D_2_O we basically measure the response of STO itself, as the OD-stretching vibration region is found to be around 2400 cm^−1^.^24^ The SFG signal observed with D_2_O in the cell is hereafter called non-resonant signal, which includes the non-resonant signal commonly observed in SFG and the contribution from STO.^17^ One can observe that the STO layer gives rise to a strong signal, the amplitude and shape of which depend on the sample position. At certain positions this signal is even strongly frequency dependent. We hypothesise that the diversity of crystallinity in the thickness of the STO layers may account for the position dependence of the STO signal. The use of a flow cell ensures that experiments in a series are performed at fixed positions. However, the measured signals with H_2_O in the cell are clearly different, especially between 3000 and 3600 cm^−1^, from the experiments with D_2_O. The signal for H_2_O pH 3 is above the signal for D_2_O in the 3200–3600 cm^−1^ region independent of the position. Moreover, as shown in Fig. 2(b), the signal for H_2_O pH 11 is above the signal for D_2_O in the 3000–3200 cm^−1^ region and below the signal for D_2_O in the 3200–3600 cm^−1^ region.

The observation that the signal for H_2_O and the signal for D_2_O in the cell are different in these regions shows that hydrogen-bonded OH-stretching vibrations contribute to the SFG signal: the observed signals arise from both the STO response and OH groups. To check that the STO response is not composed of signals from water molecules trapped in the STO layer, we performed experiments in the 2200–3000 cm^−1^ region as well. In this region, the infrared beam is not in resonance with any OH vibrations. We also observed that the STO layer gives rise to a strong SFG signal, *cf.* ESI† Fig. S3. A similar SFG signal is observed for the STO–air interface.

**Fig. 3:**
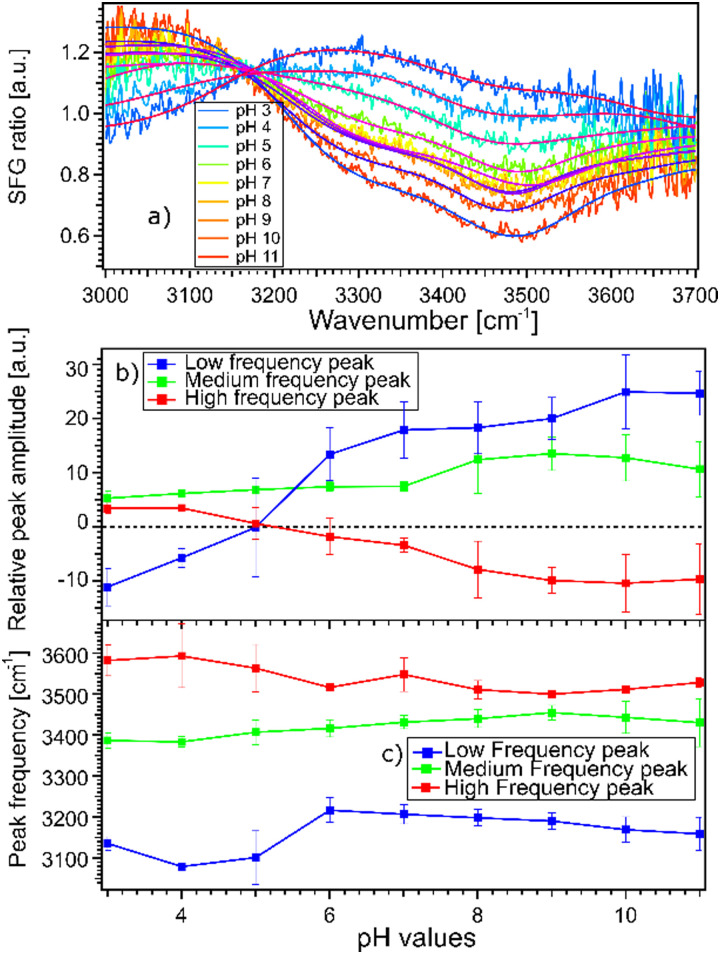
(a) SFG spectra for the STO–water pH 3 to pH 11 interface, normalized by the STO–D_2_O interface, at position 2 on the layer together with the fitting curves obtained by the Lorentzian lineshape model. Here position 2 is chosen as a representative position. (b) Amplitude of each peak obtained from fitting pH dependent SFG-spectra acquired at 3 different positions. (c) Central frequency of each of the three peaks as a function of pH. The error bars indicate the standard deviation of the three datasets.

The fact that the signal for STO–D_2_O is strong and frequency dependent is challenging because the signal from the interfacial water molecules might interfere with the signal from the STO layer itself. To be able to extract the SFG response of the water molecules, we divide the signals obtained from the STO–H_2_O interface with the STO–D_2_O interface. Fig. 3(a) shows the results for pH 3 (blue) and pH 11 (red) from Fig. 2(b) together with intermediate pH data. Below 3150 cm^−1^, the signal for water pH 3 is below the signal for water pH 11 and in the region 3200–3700 cm^−1^ and the signal for water pH 11 is below the signal for water pH 3. One can observe that all other pH values are distributed between those two signals.

Moreover, we observe that in the region 3200–3700 cm^−1^, the signals for water pH 3 to pH 6 are decreasing rapidly whereas the signals for water pH 7 to pH 9 seem to be similar. Furthermore, the signals for water pH 10 and 11 decrease in the same region. To quantify those changes, we have fitted the data using the following model.

As we divide the water spectrum by the heavy water spectrum recorded at the same position, the so-obtained intensity can be expressed as follows:1
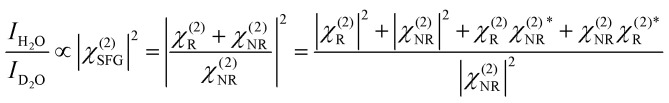

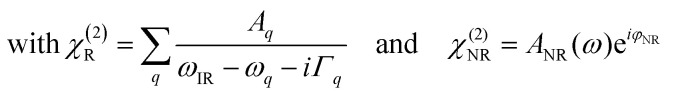
where *χ*^(2)^_R_ is the second-order non-linear susceptibility of the resonant contribution, in our case the sum of the OH-stretching vibration modes. *A*_*q*_, ω_*q*_ and *Γ*_*q*_ are the amplitude, resonant frequency and linewidth of the *q* molecular vibrational mode, respectively. *χ*^(2)^_NR_ is here defined as the second-order susceptibility of both the STO layer plus the non-resonant signal usually observed in SFG spectroscopy. It is defined by its amplitude *A*_NR_ and phase *φ*_NR_.

Experimentally, we observe that the Im(*χ*^(2)^_SFG_) and the Re(*χ*^(2)^_SFG_) and thus *A*_NR_ and *φ*_NR_ obtained for D_2_O in the cell are similar for pD 3 and pD 11, *cf.* ESI† Fig. S4. As such, we use the SFG spectra for STO-D_2_O as a pH-independent *χ*^(2)^_NR_ but dependent on the sample position. The amplitude *A*_NR_(*ω*) of the STO response is defined as the square root of the SFG spectra for STO–D_2_O, see the black line in Fig. 2(b). The phase *φ*_NR_ is used as a global fit parameter; we cannot use the phase-resolved experiments as the position on the sample is different. For the resonant contribution, we use three complex Lorentzians with pH independent full width of half maximum of 316 cm^−1^, 253 cm^−1^ and 218 cm^−1^ respectively for the lower, medium and high frequency peaks. The resulting fits are depicted in Fig. 3(a) and the fit parameters in Table S2 in the ESI.† Each spectrum could be described very well. Spectra of pH series performed at sample positions 1 and 3 can be found in ESI,† Fig. S5 and S6 with the fitting parameters in Tables S1 and S3 (ESI†).

**Fig. 4:**
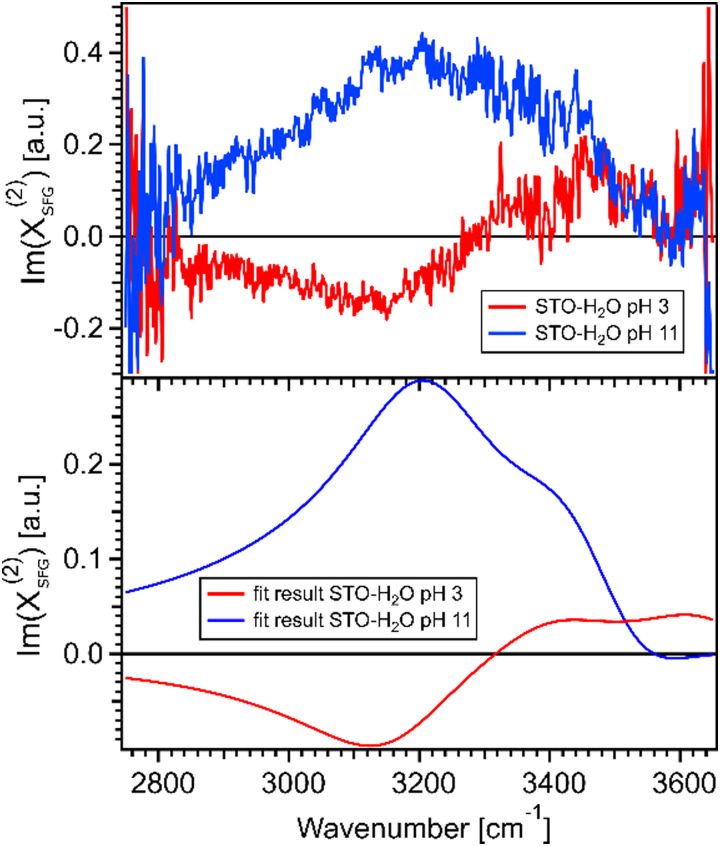
(a) Imaginary parts of phase resolved SFG spectra for water pH 3 and pH 11, normalized with D_2_O, in contact with a 100 nm SrTiO_3_ layer. (b) Imaginary parts of the fits for water pH 3 and water pH 11, data from Fig. 3(a).

Fig. 4(a) shows the imaginary part of the SFG signal *χ*^(2)^_SFG_ measured for water pH 3 and water pH 11 in contact with the STO layer; see Section 1.1 for experimental details. The imaginary part of the water pH 3 and water pH 11 signals have opposite signs in the region 2900–3300 cm^−1^ and similar signs in the region 3300–3600 cm^−1^. A positive (negative) sign in the *χ*^(2)^_SFG_ for an O–H stretching mode means that the H is pointing up towards the interface (pointing down to the bulk). Fig. 4(b) shows the imaginary part of the resulting fit, eqn (1), obtained for water pH 3 and water pH 11 from Fig. 3(a). This agreement between the imaginary part of the SFG signal obtained experimentally and obtained using the resulting fit supports the validity of the fitting procedure used to quantify the SFG spectra.

Fig. 3(b) and (c) show the amplitudes and frequencies of the three complex Lorentzians as a function of pH. The reported values are given by the average obtained by fitting the spectra at the three sample positions. The three complex Lorentzians are referenced as low, medium and high frequency peaks and are assigned to the 3200, 3400 and 3550 cm^−1^ frequencies. Fig. 3(b) shows that the high and the low frequency peaks have opposite amplitudes for all pH and they respectively flip their sign around pH 5. A change in amplitude sign suggests a change in the orientation of the dipole moment of the water species. Therefore, the changes of amplitude for the low and high frequency peaks upon changing the pH could suggest that the surface charge flips due to protonation and deprotonation of the STO layer. In the case of the medium frequency peak, its amplitude is positive over the full range of probed pH values.

Fig. 3(c) shows the pH-dependence of the central frequency of each peak. It can be seen that the frequencies are clearly separated from each other. The central frequency of each peak is determined by the average hydrogen bonding strength of the ensemble of vibrating OH-groups. The stronger the hydrogen bond of an OH group is, the lower the oscillation frequency.^28^ At pH 3 the low frequency peak has a frequency of 3120 cm^−1^, with increasing pH shifting to a lower frequency reaching a minimum around pH 5. Between pH 5 and pH 6 coinciding with the flip in orientation the frequency jumps to 3200 cm^−1^ followed by a weak decrease with increasing pH. The high frequency peak has a frequency of 3600 cm^−1^ for water at pH 3 shifting to a lower frequency reaching 3500 cm^−1^ in the case of water at pH 11. For both the low and high frequency peaks, the error bars increase around water at pH 5 related to the flip of the sign of their amplitudes around this pH value. The middle frequency peak seems to be relatively pH independent with a frequency of 3400 cm^−1^ for water at all pH values. One can conclude that the shift in frequency of all peaks is relatively small, particularly for the middle peak; therefore, their weak frequency shifts suggest a rather pH independent hydrogen bond strength.

As previously discussed the pH-dependent amplitude changes for the low and high frequency peaks could be indicative of the presence of surface charges originating from protonation or deprotonation of the STO surface. According to the work of Jan Schaefer *et al.*, the presence of charges at an interface results in a direct current (DC) field reaching into the water that can contribute to the orientation or the polarisation of the water molecules and thus to the SFG signal intensity.^29,30^ If this field becomes strong enough, it can interact with the incoming beams giving rise to a third-order response of the water molecules. In the case of a charged interface one can express the intensity of the SFG signal as2
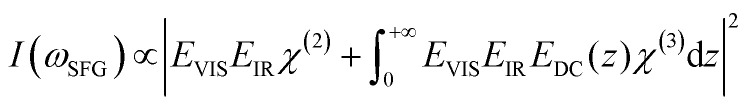
with *E*_DC_ being the strength of the DC field along the *z* axis in the direction perpendicular to the interface and *χ*^(3)^ being the third-order susceptibility of the water molecules.^29^ This process can occur for both centrosymmetric and non-centrosymmetric media. Therefore, it could give rise to a not negligible bulk contribution that can overshadow the *χ*^(2)^ signal from the direct interfacial water.^29–31^ In our case, to evaluate which part of the SFG signal rises from the interfacial layer and from the bulk contribution, one can screen the DC field by adding excess ions into the solutions. For positively charged interfaces, negatively charged ions will be attracted towards the interface, shielding the DC field and consequently decreasing the *χ*^(3)^ contribution. In the opposite scenario, for a negatively charged interface, positively charged ions will migrate towards the interface screening the bulk signal.

**Fig. 5:**
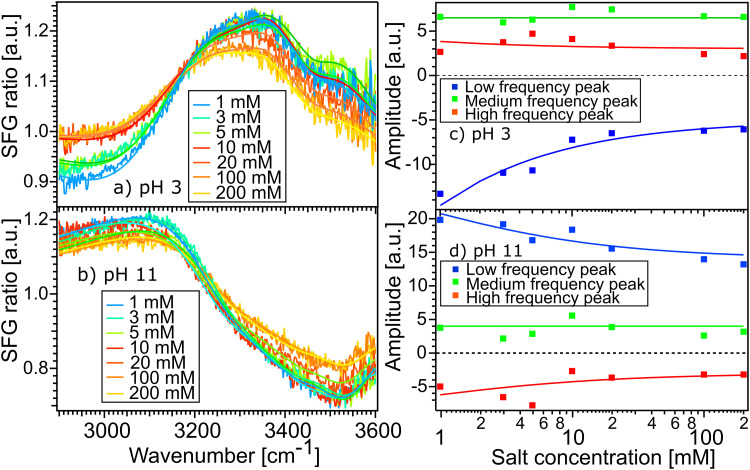
SFG spectra of H_2_O solutions of pH 3 (a) and pH 11 (b), with different NaCl concentrations, in contact with the STO layer. Amplitude of each peak obtained from fitting the SFG-spectra of water at pH 3 (c) and pH 11 (d), *versus* the ionic strength. The solid lines depict the data modelling based on the Gouy–Chapman model.

Fig. 5(a) and (b) show the SFG spectra of the STO layer in contact with H_2_O solutions of pH 11 and pH 3, with different ionic strengths of 1, 3, 5, 10, 20, 100 and 200 mM. The Debye-lengths for these salt concentrations are 9.6, 5.5, 4.3, 3.0, 2.1, 1.0 and 0.7 nm, respectively.^31^ In both Fig. 5(a) and (b), the water with the highest salt content is plotted in yellow and the water with the lowest salt content is in blue. Here, by normalizing the spectra with the STO–D_2_O spectrum, an SFG ratio of 1 indicates that there are no contributions from water molecules to the signal. In Fig. 5(a), we observe that in the 2800–3200 cm^−1^ region, upon addition of salt, the spectra of water pH 3 converge to an SFG ratio of 1, whereas in the 3200–3600 cm^−1^ region, the spectra decrease only partially towards 1. The spectra of water pH 11 are hardly affected by the addition of salt (Fig. 5(b)). To quantify those changes, we have fitted the data using eqn (1) as previously. The data are well described upon changing the resonance amplitudes of the three Lorentzians and by keeping their frequencies and their linewidths fixed. Fig. 5(c) and (d) show the amplitudes from the fit of each spectrum *versus* the concentration of salt. For both pH 3 and pH 11, the low frequency peak is significantly affected by the addition of salt in the solutions: by adding 200 mM of salt, its amplitude is reduced by half. A similar decrease of amplitude for the high frequency peak in both acidic and alkaline conditions is observed. However, the medium frequency peak seems to be independent of the salt concentration in the solutions.

Furthemore, the solid lines in Fig. 5(c) and (d) show the data modelled using the Gouy–Chapman model. This model describes the observed SFG intensity with the contribution of a constant *χ*^(2)^ contribution and a *χ*^(3)^ contribution dependent on the surface potential *ϕ*_0_ generated by the surface charge:^19,32,33^3
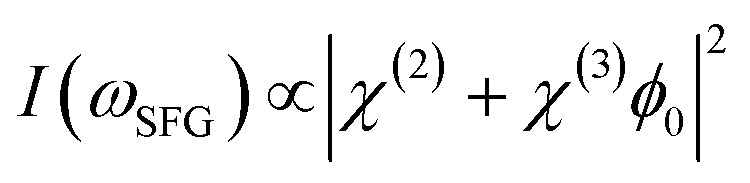
With *ϕ*_0_ being proportional to the arcsin of the surface charge density *σ* inversely proportional to the ionic strength *I* of the aqueous phase:4
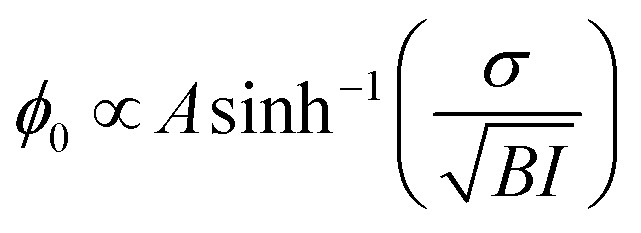
where *A* and *B* are two constants. One can find the complete description of the Gouy–Chapman model elsewhere.^19,32,33^ In our case, the surface charge density of STO has been estimated to be 0.05 C m^−2^ for pH 3 and −0.2 C m^−2^ for pH 11. The estimate is based on TiO_2_ for which the charge density is well characterised for each pH value.^34–36^ In the modelling of the middle frequency peak for both pH 3 and pH 11, we assume that the OH stretch amplitude originates solely from the *χ*^(2)^ component, we set the *χ*^(3)^ component in eqn (3) to zero. In the case of the low and high frequency, the signals are a combination of *χ*^(2)^ and *χ*^(3)^ components. In detail, at pH 11 the low frequency peak is modelled considering an equal contribution of the *χ*^(2)^ and *χ*^(3)^ components and at pH 3 the model considers the signal to be dominated by the *χ*^(3)^ components, about 10 times higher than those of *χ*^(2)^. In the case of the high frequency peak, the model also uses a *χ*^(3)^ components higher than *χ*^(2)^ in both cases of pH 3 and pH 11. As such we observe that the Gouy–Chapman model can describe very well the salt dependency data and we have observed that both low and high frequency peaks originate from a combination of *χ*^(2)^ and *χ*^(3)^ susceptibility, whereas the medium peak originates mainly from *χ*^(2)^.

In order to assign the low, medium and high frequency peaks to water species present at the interface, we first have to unravel if they arise from intra- and/or intermolecular coupling of vibrational modes. Intramolecular coupling can split one peak into a double-peak feature and the intermolecular coupling can induce a significant red-shift in the O–H stretch response.^37^ Experiments performed with isotopic dilution of water and heavy water allow us to monitor whether vibrational coupling contributes to the signals.^38^ If no molecular couplings are contributing, the amplitude of the resonant OH species will decrease linearly with the addition of OD species. If any intra- and/or intermolecular coupling of water molecules are occurring at the interface the amplitude of the resonant OH species would decrease non-linearly with the addition of heavy water. This is due to the uncoupled nature of the OH and OD stretching modes in HOD molecules, the intramolecular coupling of water molecules will be rapidly cancelled and due to the increase of distance between OH species the intermolecular coupling of water molecule will also be cancelled.^38^

**Fig. 6:**
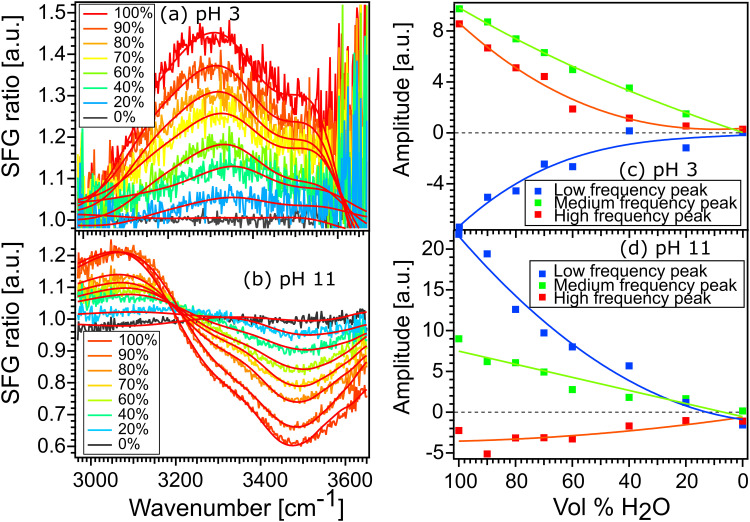
Spectra of 100% H_2_O and diluted solutions of pH 3 (a) and pH 11 (b) in contact with the STO layer and the fitting curve obtained by Lorentzians fit. Real amplitude obtained from fitting the SFG-spectra for isotopic solution of water at pH 3 (c) and pH 11 (d).

Fig. 6(a) and (b) show the spectra of 100% H_2_O and diluted solutions of pH 3 and pH 11 in contact with the STO layer. In both plots, the signal for 100% H_2_O is plotted in red and the signal for solution with 100% D_2_O is plotted in black. Here as the spectra are normalised with D_2_O the solution with 100% the D_2_O signal defines the reference spectra with an SFG ratio of 1. We observe that upon isotopic dilution the intermediate solutions for water pH 3 and water pH 11 all converge to the reference spectra. In order to quantify the changes, we have fitted the data using, as previously, three complex Lorentzians: upon changing the amplitude of the three peaks each spectrum could be described very well, for all fits the width and the frequency of each peak are fixed.

Fig. 6(c) and (d) show the amplitude of each peak *versus* the concentration of H_2_O. Upon increasing the percentage of D_2_O the amplitude of the middle peak is almost linearly reduced from 100% H_2_O to 100% D_2_O in both pH 3 and pH 11 environments. This is expected in the case when no intra- and/or intermolecular coupling is involved.

In the case of the high and the low frequency peaks, their amplitudes reduce non-linearly from 100% H_2_O to 100% D_2_O in both acidic and alkaline conditions. One can therefore conclude that they are affected by inter and/or intramolecular coupling of the water molecules. However, as both low and high frequency peaks have opposite signs, we conclude that these two peaks do not originate from the same vibration. To conclude, the middle frequency peak arises clearly from a single O–H vibration not affected by any inter- and intramolecular coupling. The low and high frequency peaks arise from two different vibrational modes which are weakly affected by intermolecular vibrational coupling.

**Fig. 7:**
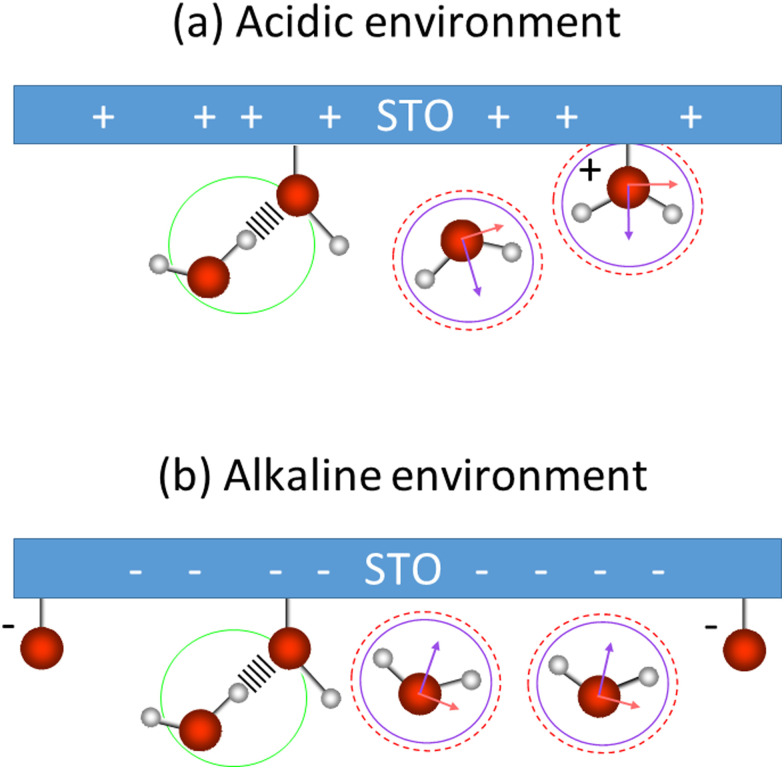
Water orientation at the interface with SrTiO_3_ depending on pH, for the acidic (a) and the alkaline (b) environments. Circles refer to the vibrational frequency (purple: low frequency; green: middle frequency; dashed red: high frequency). Arrows indicate the transition dipole moment of the symmetric (purple) and the asymmetric (red) stretching modes of water.

These low and high frequency peaks have their frequencies centred around 3150 and 3550 cm^−1^. According to the literature those frequencies can be assigned to the symmetric (low frequency peak) and the asymmetric (high frequency peak) stretching modes of water molecules.^21^ Such vibrational modes have opposite sign in the Im*χ*^(2)^_SFG_^ ^^39,40^ and thus an opposite sign in the amplitude of the Lorentzian in our fit as well. This is consistent with our results. Fig. 7 summarizes the water conformation at the interface with STO in acidic (a) and alkaline (b) environments: the symmetric and asymmetric water stretching modes are depicted respectively by purple and dashed red marks. In an acidic environment, H_2_O^+^ groups are likely to be present at the interface as a result of H_2_O molecules chemisorbed onto the STO layer or to protonated OH chemisorbed groups. The interfacial model, based on the literature,^11,15^ describes the presence of H_2_O, Ti–OH and H_2_O^+^ at the STO–water interface. Moreover, we propose the presence of interfacial O^−^ in alkaline conditions accounting for deprotonation sites.

Regarding the middle frequency peak, the sign of the amplitude does not vary with the pH value of the aqueous solution. Its positive amplitude indicates an OH species pointing with the hydrogen toward the STO surface. In the case of STO, it is expected that water desorbs on TiO_2_-terminated STO surfaces.^12,13^ Yet we know that, the second layer of hydration in contact with TiO_2_ is composed of water molecules directed toward the surface.^41^ Therefore, we assign the middle frequency peak to weakly hydrogen-bonded OH groups oriented towards the STO layer, indicated by a green mark in Fig. 7. Additionally, as described by isotopic dilution, it is unaffected by any inter- or intramolecular coupling. As such we conclude that, the frequency of this O–H group in the water molecule significantly differs from the other half of the molecule, preventing any coupling from occurring. This O–H stretching vibration of the other half of the molecule has most likely a vibrational frequency underlying one of the others in our analysis and as such cannot be specifically assigned.

In contrast to previous SFG work studying the TiO_2_–water interface,^24,26^ we do not seem to be sensitive to chemisorbed Ti–OH species. Ti–OH groups would be pointing with their hydrogen atom away from the surface, but in our fitting results we could not observe peaks with an according sign for the amplitude. However, such species might well be present, but in minority, in the spectra. In the aforementioned SFG works,^24,26^ OH groups of physisorbed water molecules at 3100 cm^−1^ oriented with the hydrogen towards the TiO_2_ surface were observed. However, in our case at the interface with STO, the physisorbed OH-groups are observed at a lower frequency (higher wavenumber). This difference can be explained by the fact that STO does not have superhydrophilicity^42,43^ in contrast to TiO_2_,^44^ causing the physisorbed OH groups to be weaker hydrogen bonded to STO surfaces. The frequency of these O–H groups at 3400 cm^−1^ is comparable to the frequency of the H-bonded water at the water/air interface.^45^ Moreover, in our case the use of a polycrystalline STO layer presents a complex situation^46^ where the different crystalline facets and the presence of strontium atoms in the bulk might also affect the hydrogen bonding strength of the OH groups.

One can interpret the change of sign of the symmetric and asymmetric stretching modes of water at pH 5 as the point of zero charge of the STO layer. As shown in Fig. 3, we observe an intensity-minimum at around pH 5, corresponding to the balance of protonation and deprotonation of STO. At this point, the water molecules are randomly oriented leading to a weak SFG signal. This result is in agreement with a charged titanium dioxide water interface as reported by SFG spectroscopy showing a point of zero charge close to pH 5.^24,25^ Therefore, this observation is in line with previous STO water interaction studies presenting STO as a TiO_2_ terminated surface.^11–16^ However, as mentioned earlier, we observed that physisorbed OH groups are more weakly hydrogen bonded to the surface.

## Conclusions

The SrTiO_3_–H_2_O interface has been probed for a polycrystalline 100 nm layer by SFG spectroscopy for pH values from 3 to 11. After normalization by the non-resonant response, the spectra show three different peaks assigned to symmetric and asymmetric stretching modes of physisorbed water molecules and to water molecules donating a H-bond to probably surface TiOH groups. For all pH values, the species responsible for those peaks are the same and their hydrogen bonding environment does not change significantly. The spectra show a minimum peak intensity at pH 5 close to the point of zero charge of the TiO_2_ surface. The amplitude of the physisorbed water peaks flip signs upon crossing the point of zero charge indicating a reorientation of the water groups due to the protonation and deprotonation of the STO layer.

## Conflicts of interest

There are no conflicts to declare.

## Supplementary Material

CP-025-D3CP03829G-s001
