# Supplementary material for: Unravelling the interfacial water structure at the photocatalyst strontium titanate by sum frequency generation spectroscopy
Source: Phys Chem Chem Phys. 2023 Oct 31;25(45):31471–80. doi: 10.1039/d3cp03829g (PMC10664186; doi:10.1039/d3cp03829g)
Supplement: CP-025-D3CP03829G-s001 [file CP-025-D3CP03829G-s001.pdf]

Electronic Supplementary Material (ESI) for Physical Chemistry Chemical Physics.
This journal is © the Owner Societies 2023

Supplementary Information

Unravelling the interfacial water structure at the photocatalyst strontium titanate by sum frequency generation spectroscopy

Martin Buessler[1,2], Shingo Maruyama[3], Moritz Zelenka[1,2], Hiroshi Onishi[4,5], Ellen H.G. Backus[1,2]

[1] University of Vienna, Faculty of Chemistry, Institute of Physical Chemistry, Währinger Straße 42, 1090 Vienna, Austria
[2] University of Vienna, Vienna Doctoral School in Chemistry (DoSChem), Währinger Straße 42, 1090 Vienna, Austria
[3] Department of Applied Chemistry, Graduate School of Science, Tohoku University, Sendai, Miyagi, Japan
[4] Department of Chemistry, School of Science, Kobe University, Rokko-dai, Nada, Kobe, Japan
[5] Division of Advanced Molecular Science, Institute for Molecular Science, Myodaiji, Okazaki, Japan

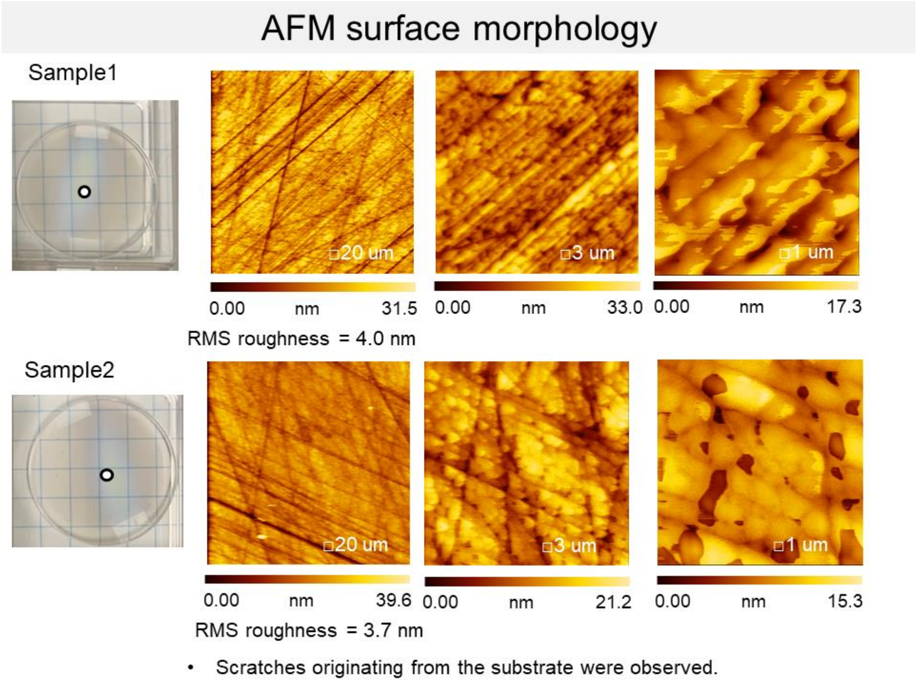

Figure S1. Atomic force microscope (AFM) surface morphology of the CaF_2-STO samples. The sample number 1 has been used to carry out all the measurements of this study.

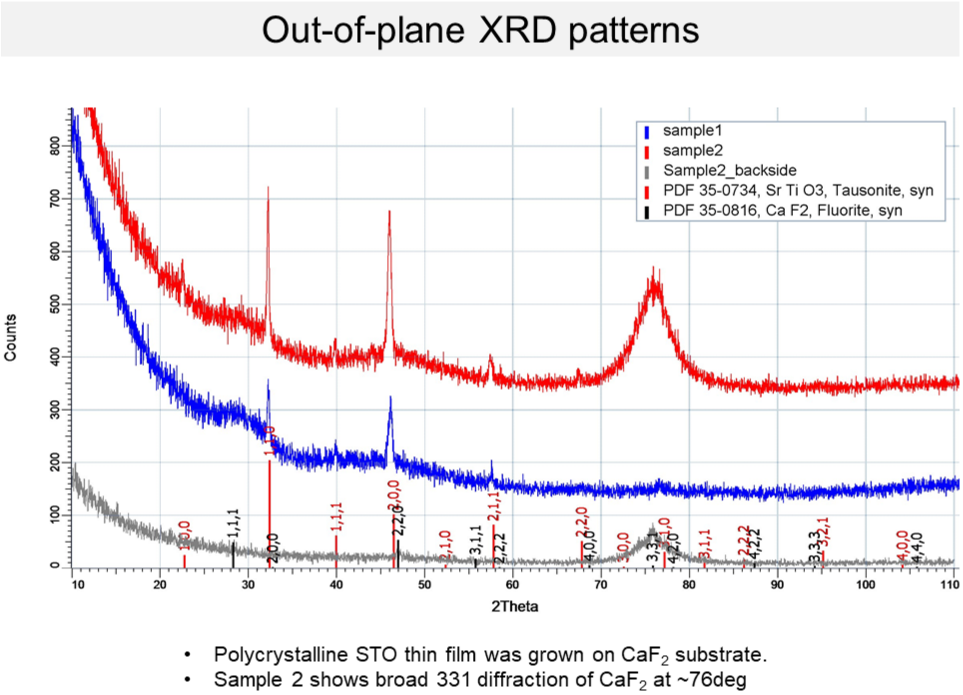

Figure S2. X-ray diffraction (XRD) spectra of the CaF_2-STO samples. The sample number 1 has been used to carry out all the measurements of this study.

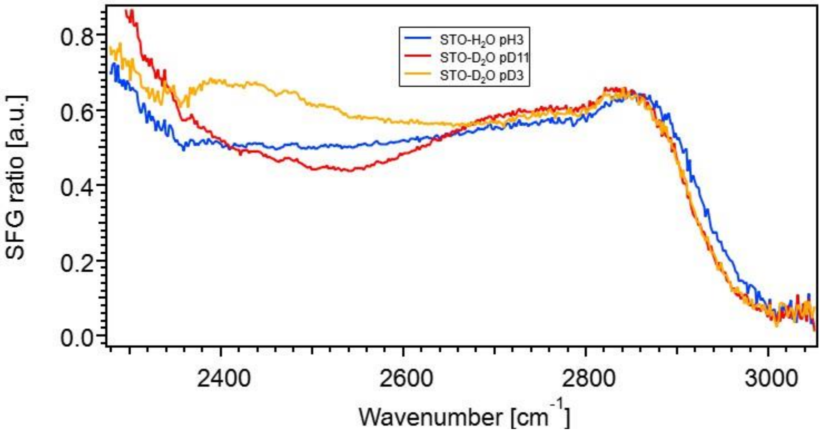

Figure S3. Spectra for water pH 3 and D_2O pD 3 and pD 11, normalized with gold, in contact with a 100 nm $SrTiO_3$ layer. The experiment is performed in the O-D stretch vibrations region, thus here the resonant contribution of our signal is given by the O-D vibration and the signal from water at pH 3 corresponds to the STO response. Even in this frequency region, the strontium titanate layer gives rise to a strong non-resonant signal.

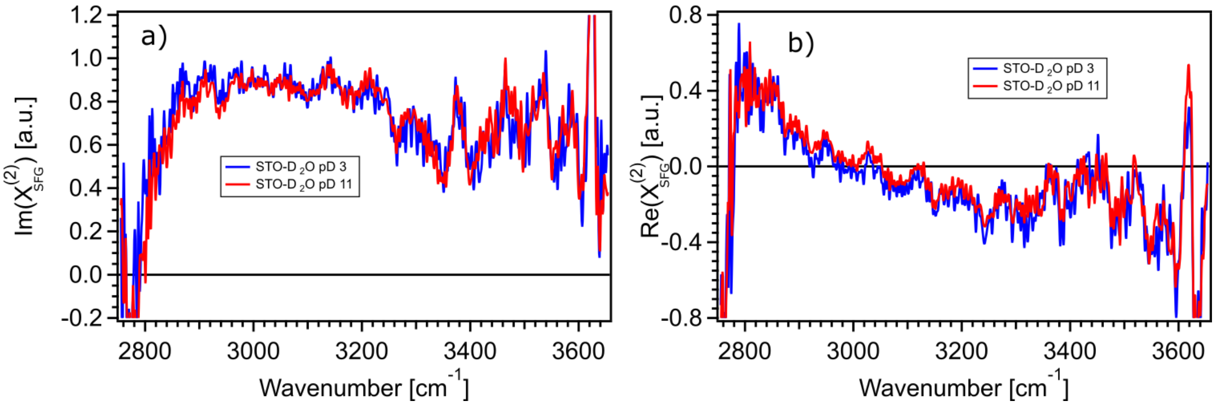

Figure S4. Phase resolved spectra for water pD 3 and water pD 11, normalized with gold, in contact with a 100 nm SrTiO3 layer. Here $\chi^{(2)}_{SFG} = \frac{\chi^{(2)}_{R}+\chi^{(2)}_{NR}}{\chi^{(2)}_{NR}}$ *(a) Spectra of the imaginary part of* $\chi^{(2)}_{SFG}$ *, (b) Spectra of the real part of* $\chi^{(2)}_{SFG}$.

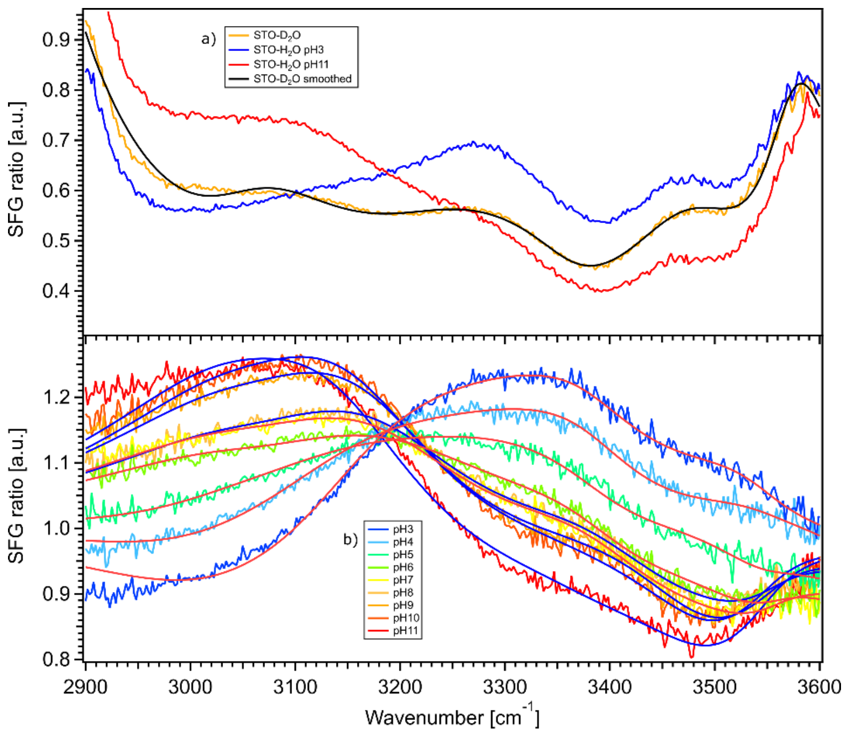

Figure S5. (a) Spectra for pure D_2O (pD 7), water pH 3 and water pH 11, normalized with gold, in contact with a 100 nm $SrTiO_3$ layer at position number 1 on the sample. The smoothed STO-D_2O curve is depicted in black (=$A_{NR}(\omega)^2$).
(b) Spectra for water pH 3 to water pH 11, normalized with D_2O, in contact with a 100 nm $SrTiO_3$ layer at position number 1 on the layer together with fits with the Lorentzian model.

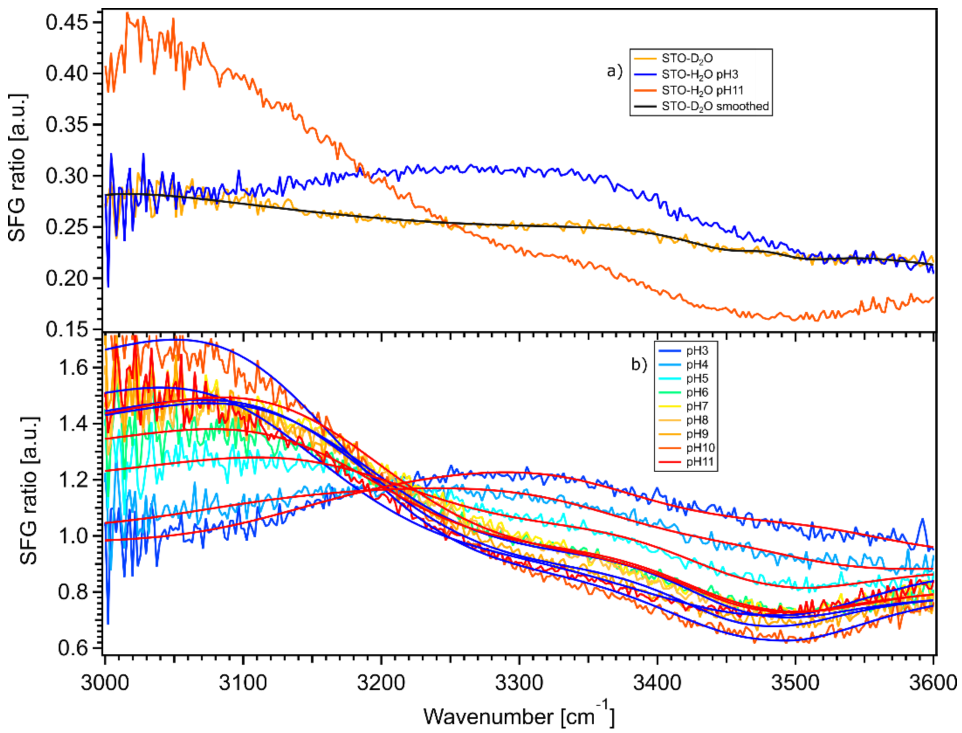

Figure S6. (a) Spectra for pure D_2O (pD 7), water pH 3 and water pH 11, normalized with gold, in contact with a 100 nm $SrTiO_3$ layer at position number 3 on the sample. The smoothed STO-D_2O curve is depicted in black (=$A_{NR}(\omega)^2$).
(b) Spectra for water pH 3 to water pH 11, normalized with D_2O, in contact with a 100 nm $SrTiO_3$ layer at position number 3 on the layer together with fits with the Lorentzian model.

Table S1. Fitting parameters used for SFG measurement at position 1 depicted in Figure S5 (b). The NR amplitude is obtained from the square root of the STO-D_2O signal depicted in Figure S5 (a).

	pH11	pH10	pH9	pH8	pH7	pH6	pH5	pH4	pH3
NR phase	3,78	3,89	3,70	3,59	3,37	3,19	3,07	3,32	3,36
Amplitude 1	19,32	17,65	16,49	11,90	11,40	7,42	-4,15	-7,95	-14,35
Frequency 1	3145,35	3160,02	3182,90	3209,41	3220,89	3247,47	3052,99	3083,07	3124,70
Width 1	316	316	316	316	316	316	316	316	316
Amplitude 2	15,97	16,63	11,57	5,42	6,52	6,16	6,59	6,84	6,10
Frequency 2	3497,81	3487,46	3468,67	3419,65	3417,18	3391,27	3367,20	3385,11	3379,21
Width 2	253	253	253	253	253	253	253	253	253
Amplitude 3	-18,95	-17,68	-10,54	-2,96	-1,64	2,69	4,18	3,82	4,68
Frequency 3	3513,75	3506,55	3509,11	3541,15	3598,30	3507,28	3526,83	3586,19	3558,58
Width 3	218	218	218	218	218	218	218	218	218

Table S2. Fitting parameters used for SFG measurement at position 2 depicted in Figure 3 (a). The NR amplitude is obtained from the square root of the STO-D_2O signal depicted in Figure 2 (a).

	pH11	pH10	pH9	pH8	pH7	pH6	pH5	pH4	pH3
NR phase	2,88	2,94	3,02	3,08	3,10	3,22	2,13	2,93	2,82
Amplitude 1	29,24	22,88	18,03	19,37	18,06	13,31	-8,80	-5,74	-12,84
Frequency 1	3214,39	3210,93	3217,62	3214,98	3225,87	3226,30	3057,20	3080,64	3158,05
Width 1	316	316	316	316	316	316	316	316	316
Amplitude 2	12,05	14,87	17,90	20,41	7,28	7,93	6,82	5,93	3,42
Frequency 2	3436,60	3449,65	3465,18	3469,94	3453,63	3442,82	3438,34	3397,46	3411,21
Width 2	253	253	253	253	253	253	253	253	253
Amplitude 3	-5,68	-8,84	-12,63	-15,16	-4,36	-5,28	0,46	3,53	2,73
Frequency 3	3543,64	3502,88	3500,09	3501,42	3546,08	3521,45	3645,06	3690,13	3634,42
Width 3	218	218	218	218	218	218	218	218	218

Table S3. Fitting parameters used for SFG measurement at position 3 depicted in Figure S6 (b). The NR amplitude is obtained from the square root of the STO-D_2O signal depicted in Fig. S6 (a).

	pH11	pH10	pH9	pH8	pH7	pH6	pH5	pH4	pH3
NR phase	3,63	3,47	3,34	3,36	3,44	3,34	3,49	3,00	3,39
Amplitude 1	25,44	34,21	25,41	23,62	24,13	19,34	12,46	-3,70	-6,37
Frequency 1	3116,77	3135,85	3169,89	3169,89	3173,27	3175,94	3192,99	3073,63	3123,34
Width 1	316	316	316	316	316	316	316	316	316
Amplitude 2	3,77	6,71	11,05	11,27	8,50	8,03	7,06	5,58	6,18
Frequency 2	3355,42	3392,08	3429,60	3429,60	3423,17	3415,04	3414,60	3367,06	3368,58
Width 2	253	253	253	253	253	253	253	253	253
Amplitude 3	-4,52	-4,98	-6,78	-5,66	-4,41	-3,00	-2,89	2,90	2,65
Frequency 3	3527,06	3524,21	3489,67	3489,67	3497,87	3519,24	3517,26	3501,31	3551,40
Width 3	218	218	218	218	218	218	218	218	218
